# The human papillomavirus E6 and E7 inducible oncogene, *hWAPL*, exhibits potential as a therapeutic target

**DOI:** 10.1038/sj.bjc.6602329

**Published:** 2005-01-11

**Authors:** M Kuroda, T Kiyono, K Oikawa, K Yoshida, K Mukai

**Affiliations:** 1 Department of Pathology, Tokyo Medical University, Shinjuku-ku, Tokyo 160-8402, Japan; 2 CREST Research Project, Japan Science and Technology Corporation, Kawaguchi-shi, Saitama 332-0012, Japan; 3 Shinanomachi Research Park, Keio University, Shinjuku-ku, Tokyo 160-8582, Japan; 4 Division of Virology, National Cancer Center Research Institute, Chuo-ku, Tokyo 104-0045, Japan

**Keywords:** hWAPL, HPV, E6, E7, siRNA

## Abstract

Here we show that human papillomavirus (HPV) E6 and E7 oncoproteins induce hWAPL expression. In addition, small interfering RNA (siRNA) of *hWAPL* suppressed the growth of tumours derived from SiHa cells in nude mice. Thus, hWAPL may be one of the effective targets of uterine cervical cancer therapy.

Cervical cancer is unique due to its association with high-risk human papilloma virus (HPV) infection with strains such as HPV-16 and HPV-18 (zur Hausen, 1996). The high-risk-HPV oncoproteins, E6 and E7, are necessary for immortalisation and transformation of cervical keratinocytes (Munger *et al*, 1989). E6 binds to the wild-type p53 protein and promotes its ubiquitin-dependent degradation (Scheffner *et al*, 1990, 1993), and E7 binds to the retinoblastoma protein Rb and disrupts the complex between Rb and the E2F transcription factor family, which controls the expression of genes involved in cell-cycle progression (Dyson *et al*, 1992). Therefore, the development of anticancer therapies that target HPV E6 and E7 and/or downstream targets of HPV E6 and E7 might be a specific and effective treatment for cervical cancer. In fact, RNA interference (RNAi) of E6 and/or E7 inhibits growth of HPV-positive cancer cells (Jiang and Milner, 2002; Butz *et al*, 2003; Yoshinouchi *et al*, 2003).

Our previous study demonstrated that the novel human gene *hWAPL* showed the unscheduled high-level expression in cervical dysplasia and carcinoma (Oikawa *et al*, 2004). In addition, NIH3T3 cells overexpressing hWAPL developed into tumours upon injection into nude mice, suggesting that hWAPL plays a significant role in cervical carcinogenesis and tumour progression as an oncogene (Oikawa *et al*, 2004). Furthermore, siRNA of *hWAPL* inhibited cell growth in cervical cancer-derived cultured cells (Oikawa *et al*, 2004).

In this report, we reveal that HPV E6 and E7 oncoproteins affect hWAPL expression. We also show that hWAPL exhibited great potential as a novel therapeutic target molecule in the treatment of cervical cancer.

## MATERIALS AND METHODS

### Cell culture, retroviral vector construction and retroviral infection

SiHa, CaSki and C33A cells were grown in DMEM (Sigma Chemical Co., St Louis, MO, USA) containing 10% fetal bovine serum (FBS) at 37°C in a 5% CO_2_ environment. Normal human epidermal keratinocytes from adult skin (HDK1) were grown in keratinocyte serum-free medium (K-SFM; Invitrogen, Carlsbad, CA, USA).

The HPV16-E6, E7, and E6E7 genes were amplified by polymerase chain reaction (PCR) using the following primers: 5′-AAAAAGCAGGCTCCACCATGTTTCAGGACCCACAGGAGCGACCC-3′ and 5′-AGAAAGCTGGGTTACAGCTGGGTTTCTCTACGTG-3′ for E6, and 5′-AAAAAGCAGGCTCCACCATGCATGGAGATACACCTACAT-3′ and 5′-AGAAAGCTGGGTTATGGTTTCTGAGAACAGATGGGG-3′ for E7. The amplified DNA fragments were cloned into the retroviral vector, pCLXSN.

The bovine papillomavirus type 1 (BPV1) E2 segment was obtained by nested PCR from a pBPV-MII template using 5′-GGGGACAAGTTTGTACAAAAAAGCAGGCT-3′ and 5′-GGGGACCACTTTGTACAAGAAAGCTGGGT-3′ as outer primers, and 5′-AAAAAGCAGGCTCCACCATGGAGACAGCATGCGAAC-3′ and 5′-AGAAAGCTGGGTCAGAAGTCCAAGCTGGCTGTAAAG-3′ as inner primers. The BPV1 E2 segment was then cloned into a pCMSCV-based (Clontech, Palo Alto, CA, USA) retroviral vector, pCMSVpuro.

The preparation of recombinant retroviruses and the infection procedures have been previously described (Naviaux *et al*, 1996). HDK1 cells were infected with the prepared retroviruses, LXSN-16E6, LXSN-16E7, LXSN-16E6E7, or the control LXSN. The infected cells were selected in the presence of 50 *μ*g ml^−1^ of G418. SiHa, CaSki and C33A cells were infected with either the MSCV-puro or MSCV-puroBPV1E2 viruses and selected with 1 *μ*g ml^−1^ of puromycin.

### Immunoblot analysis

We generated a rabbit polyclonal antibody against a synthetic peptide, amino acids 50–66 (CNFKPDIQEIPKKPKVEE) of hWAPL (termed anti-hWAPL-N). Immunoblot analyses were performed as previously described (Kiyono *et al*, 1997). Antibodies against hWAPL (anti-hWAPL-N), p53 (Oncogene Science, Cambridge, MA, USA, DO-1), and the anti-HPV16 E7 (ZYMED, South San Francisco, CA, USA, 8C9) were used at a dilution of 1/1000.

### Animals and treatments

Guidelines for the care and use of animals were approved by the animal research centre in Tokyo Medical University. BALB/cAJc1-nu female mice (4 weeks old) were purchased from Charles River Japan, Inc. (Kanagawa, Japan). The individual mice were injected with ≈4 × 10^7^ SiHa cells suspended in 200 *μ*l of PBS. Upon the development of a tumour from the injected SiHa cells 1 week after injection, we injected either an active siRNA specific for *hWAPL* or the negative control siRNA at the indicated time points (Figures 2A and 3). The nucleotide sequences of the siRNAs were as previously described (Oikawa *et al*, 2004). The active siRNA specific for *hWAPL* corresponds to siRNA(I) in the previous study (Oikawa *et al*, 2004). The injection materials were generated as follows: In Figure 2, the siRNAs were synthesised by *in vitro* transcription using a Silencer siRNA Construction Kit (Ambion, Austin, TX, USA). One microlitre of 10 *μ*M siRNA and 2 *μ*l of Oligofectamine Reagent (Invitrogen Japan, Tokyo, Japan) were diluted into 89 *μ*l and 8 *μ*l of Opti-MEM I (Invitrogen Japan), respectively. After a 10-min incubation, the diluted reagents were combined and incubated for an additional 20 min. The siRNA-Oligofectamine mixture was then injected into the mouse at the tumour site. In Figure 3, 1 *μ*l of 10 *μ*M siRNA and 74 *μ*l of 2% atelocollagen (Koken Co. Ltd, Tokyo, Japan) (Takei *et al*, 2004) were mixed on ice, and then the siRNA-atelocollagen mixture was injected into mouse at the tumour site.

### Statistical analysis

The data were analysed using the Student's *t*-test, and *P*<0.001 were considered to indicate significant differences.

## RESULTS

### HPV oncoproteins E6 and E7 induce hWAPL

**Figure 1:**
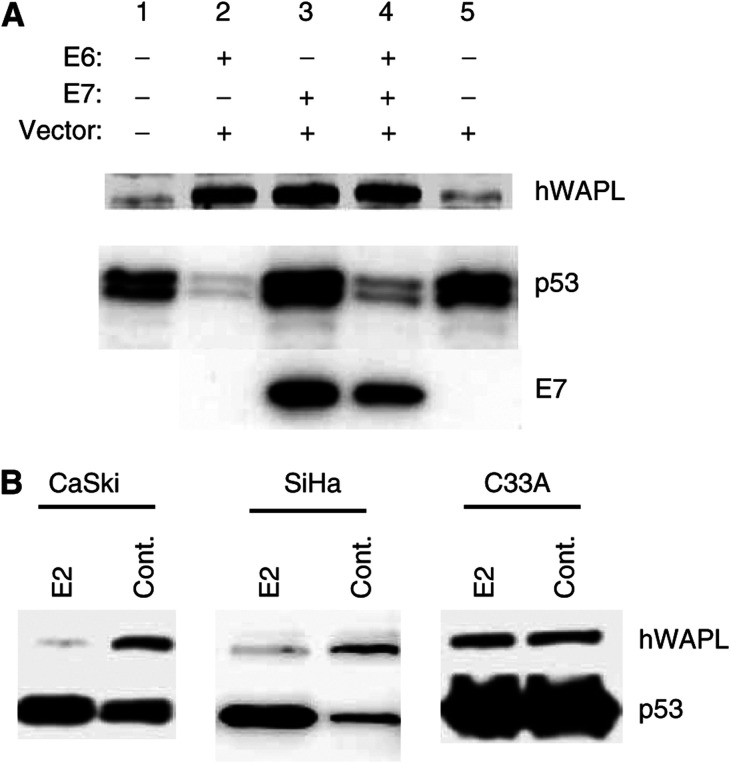
(**A**) HPV-16 E6 and E7 expression induces increased expression of hWAPL. hWAPL protein was induced by HPV E6 and E7 oncoprotein expression in HDK1 cells (passage 5), as determined by Western blotting. E6 induced p53 degradation (lanes 2, E6 and 4, E6E7). A Western blot showing HPV-16 E7 protein levels is provided in the bottom panel. (**B**) BPV1 E2 suppressed hWAPL expression. hWAPL protein levels were reduced by exogenous BPV1 E2 expression in the HPV 16 E6- and E7-expressing cell lines, CaSki and SiHa, as determined by Western blot analysis.

We examined whether HPV-16 E6 and E7 oncogene products influence hWAPL expression. We infected normal human epidermal keratinocytes from adult skin tissue (HDK1) with HPV-16 E6- and E7-expressing retroviruses, and observed that both HPV-16 E6 and E7 induce hWAPL expression (Figure 1A). E6 and E7 expression levels were confirmed by monitoring p53 degradation and E7 antibody staining, respectively (Figure 1A).

To confirm that hWAPL expression is inducible by E6 and E7, we also examined whether repression of E6 and E7 expression causes hWAPL reduction in cervical cancer-derived cell lines. Because the papillomavirus E2 protein represses E6/E7 transcription by binding the HPV early promoter (Hwang *et al*, 1993, 1996; Dowhanick *et al*, 1995; Francis *et al*, 2000), we analyse the effect of E2 on hWAPL expression. We observed a significant reduction of hWAPL protein levels after the infection of HPV-16 E6- and E7-expressing cervical cancer cell lines, such as CaSki and SiHa, with the retrovirus encoding E2 (Figure 1B). The HPV-negative cervical cancer cell line C33A, however, did not exhibit significant changes in hWAPL protein levels after E2 retrovirus infection (Figure 1B). These results demonstrate that hWAPL can be induced by HPV E6 and E7 oncoproteins.

The mechanism of hWAPL induction by E6 and E7 has not yet been elucidated. Although both E6 and E7 contribute transformation of human keratinocytes, functions of these proteins are different. hWAPL induction by the combination of E6 and E7, however, showed similar level to that by either E6 or E7 alone (Figure 1A). Thus, *hWAPL* transcription may possibly respond to a particular precancerous cell states induced by E6 and/or E7. In fact, HPV-positive normal cervical tissue samples exhibited a low level of hWAPL expression (Oikawa *et al*, 2004), and C33A, an HPV-negative uterine cervical cancer-derived cell line, showed high hWAPL expression (Figure 1B, and data not shown), suggesting that hWAPL expression is more closely related with cervical carcinogenesis than HPV infection, as previously mentioned (Oikawa *et al*, 2004). Nevertheless, although HPV infection does not always induce hWAPL expression, E6 and E7 oncoproteins are still likely to be associated with hWAPL expression in cervical cancers.

### hWAPL exhibits great potential as a therapeutic target

**Figure 2:**
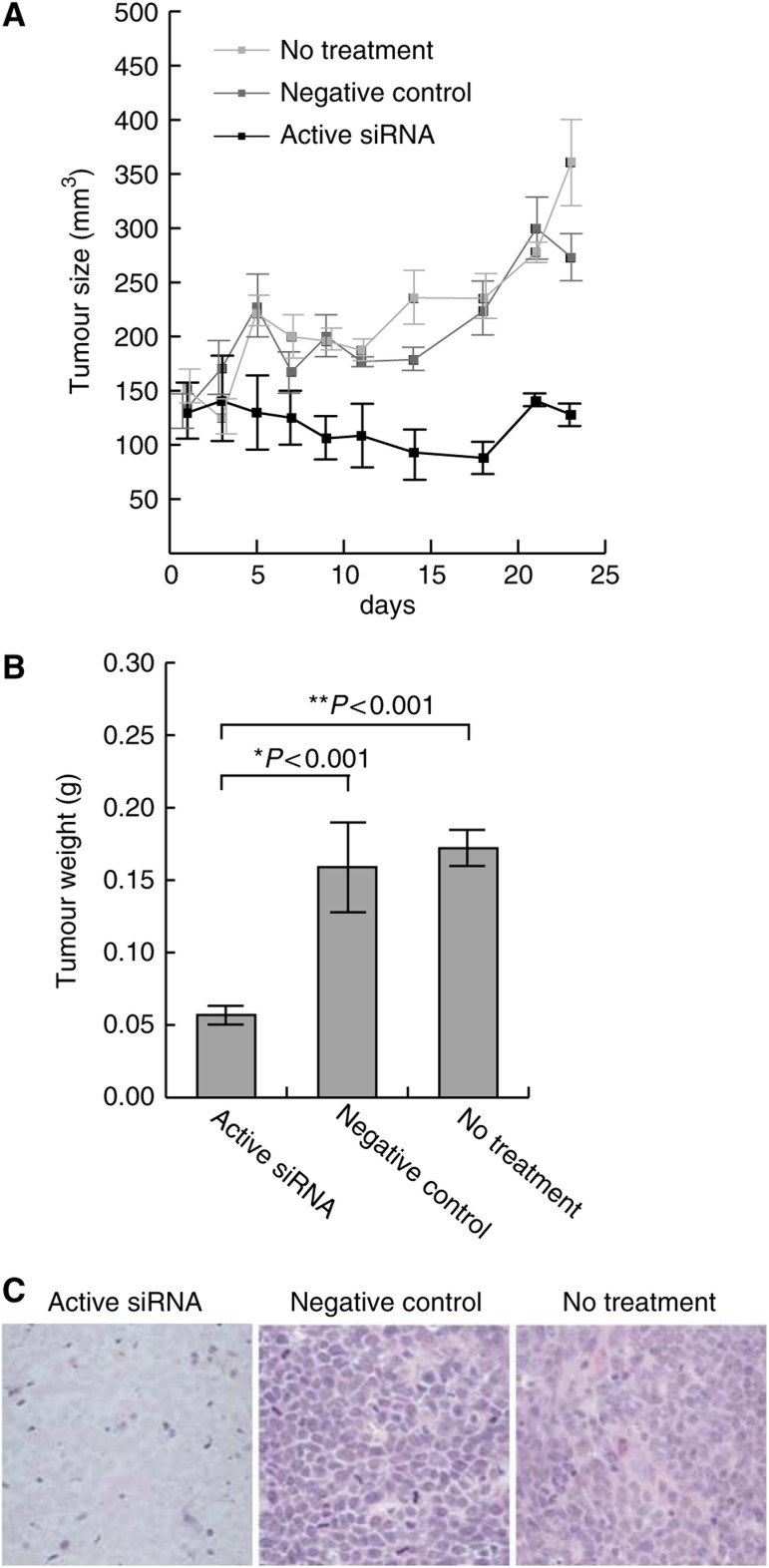
An active siRNA inhibiting hWAPL expression suppressed the growth of subcutaneous tumours derived from SiHa cells inoculated into nude mice. (**A**) Tumour growth curves. The tumour volume (mm^3^) was approximated by multiplication of the major axis, the minor axis, and the height of each tumour. Each data point represents the mean of eight samples. Bars, s.e. (**B**) Weights of the tumours in nude mice 25 days after the first siRNA injection. Each data point represents the mean of eight samples. Bars, s.e. ^*^P<0.001 *vs* negative control. ^**^P<0.001 *vs* no treatment. (**C**) Representative histology of the subcutaneous tumours in nude mice. The excised tumours were subjected to paraffin sectioning, then stained with haematoxylin and eosin.

We previously observed that repression of hWAPL expression by a specific siRNA inhibited cell growth in various cervical cancer-derived cell lines such as SiHa, CaSki (Oikawa *et al*, 2004) and C33A (data not shown) *in vitro*. Thus, we next investigated the possibility that hWAPL might be a novel cancer therapeutic target molecule. We generated tumours in nude mice by subcutaneous injection of SiHa cells. At 1 week after injection of the tumour cells, we measured tumour sizes using caliper squares and then injected siRNA into the tumours on alternate days for 3 weeks (Figure 2A). The growth of tumours injected with the active siRNA specific for *hWAPL* was repressed in comparison with untreated tumours or tumours injected with negative control siRNA (Figure 2A, B). We observed necrosis in the tumours injected with the active siRNA (Figure 2C). In contrast, untreated or control tumours were viable (Figure 2C). We also observed similar effects of the siRNA on tumours arising from another cervical cancer-derived cell line, CaSki (data not shown). These results demonstrate that hWAPL may have potential as a therapeutic target, particularly in cervical cancer.

**Figure 3:**
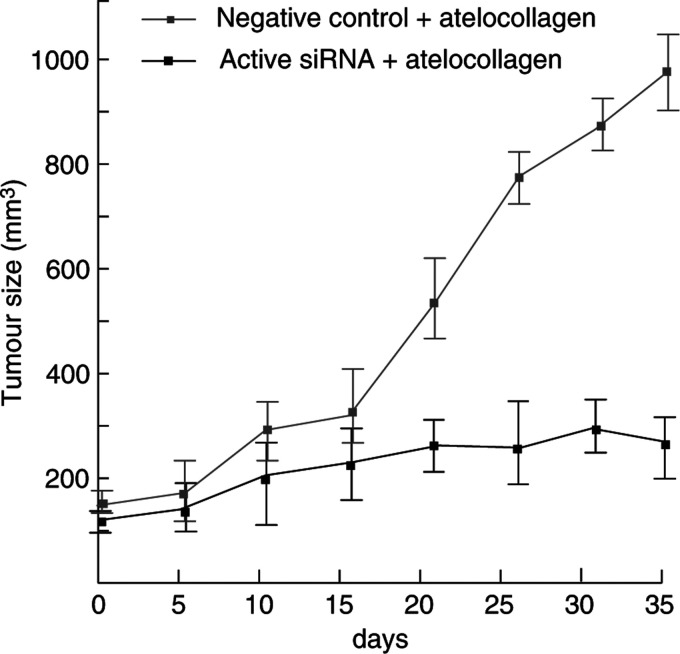
Atelocollagen increases antitumour effects of *hWAPL* siRNA. The growth curves of subcutaneous tumours injected with active or negative control siRNA mixed with atelocollagen were shown. The tumour volume (mm^3^) was approximated by multiplication of the major axis, the minor axis, and the height of each tumour. Each data point represents the mean of four samples. Bars, s.e.

However, siRNA itself is rapidly degraded in tumours (Takei *et al*, 2004) and the durability of the effects may be insufficient for clinical application. In fact, although we injected the *hWAPL* siRNA into tumours on alternate days, the repression of the tumour growth was not preserved in a few cases (Figure 2A and data not shown). Recently, a new gene transfer method using atelocollagen has been established (Ochiya *et al*, 1999). Atelocollagen is expected to increase cellular uptake, nuclease resistance, and prolonged release of siRNAs adiministered into tumours because of its unique property that it is a liquid at 4°C and a gel at 37°C (Ochiya *et al*, 1999; Takei *et al*, 2004). Furthermore, Takei *et al* revealed that atelocollagen contributes the increased stability of siRNAs injected in tumours. Thus, we generated tumours in nude mice by subcutaneous injection of SiHa cells again, and then monitored the growth of the tumours injected with active siRNA or negative control siRNA mixed with atelocollagen (Figure 3). The injection was performed at only 4 time points of 0, 5th, 10th and 20th day for 35 days. As shown in Figure 3, active siRNA with atelocollagen dramatically suppressed tumour growth.

## DISCUSSION

Recent findings show that the targeted inactivation of oncogenes could be a specific and effective treatment for cancer (Felsher, 2003). For uterine cervical cancer, HPV E6 and/or E7 were shown to be a strong candidate as a target for gene-specific therapy (Jiang and Milner, 2002; Butz *et al*, 2003; Yoshinouchi *et al*, 2003). However, many subtypes of high-risk HPV have been detected in cervical cancers although HPV types 16 and 18 are great majority of cervical carcinomas (Nakagawa *et al*, 2000). Thus, for clinical application of E6 and E7 siRNAs, various nucleotide sequences may be required for the respective types of HPV. In this study, we have demonstrated that an siRNA of *hWAPL* induces tumour regression *in vivo*. This anticancer effects by the *hWAPL* siRNA stands comparison with the effects of the *E6* siRNA (Jiang and Milner, 2002; Butz *et al*, 2003; Yoshinouchi *et al*, 2003). Therefore, we expect that hWAPL is one of strong candidates as a target for uterine cervical cancer therapy.
